# The Japanese Breast Cancer Society Clinical Practice Guidelines, 2018 edition: the tool for shared decision making between doctor and patient

**DOI:** 10.1007/s12282-019-01021-x

**Published:** 2019-11-22

**Authors:** Hiroji Iwata, Shigehira Saji, Masahiko Ikeda, Masashi Inokuchi, Takayoshi Uematsu, Tatsuya Toyama, Rie Horii, Chikako Yamauchi

**Affiliations:** 1 Department of Breast Oncology, Aichi Cancer Center Hospital, 1-1, Kanokoden, Chikusa-ku, Nagoya, Aichi 464-8681 Japan; 2 Department of Medical Oncology, Fukushima Medical University, Fukushima, Japan; 3 Department of Breast and Thyroid Surgery, Fukuyama City Hospital, Fukuyama, Japan; 4 Department of Breast Endocrine Surgery, Kanazawa Medical University, Ishikawa, Japan; 5 Department of Breast Imaging and Breast Intervention Radiology, Shizuoka Cancer Center, Shizuoka, Japan; 6 Department of Breast Surgery, Nagoya City University Graduate School of Medical Sciences, Nagoya, Japan; 7 Department of Pathology, Saitama Cancer Center, Ina, Japan; 8 Department of Radiation Oncology, Shiga General Hospital, Moriyama, Japan

**Keywords:** Guideline, Breast cancer, Japan

## Abstract

The Breast Cancer Clinical Practice Guidelines, 2018 edition, written in Japanese and organized by the Japanese Breast Cancer Society, were published in Japanese in May 2018. The process of making these guidelines, as well as the content, was largely changed and compared with previous editions. The concept of these guidelines is to act as a support tool for shared decision making between doctor and patient. The procedure of creating the guidelines referred to Minds Handbook for Clinical Practice Guideline Development 2014. This guideline, written in Japanese, consists of two booklets: (1) the epidemiology and diagnosis booklet covering screening, radiological, and pathological diagnosis and (2) the treatment booklet covering surgical therapy, radiation therapy, and systemic therapy. This review article consists of five parts, including the history of the Breast Cancer Clinical Practice Guidelines, the concept, process, content, and recommendation grade. I believe this brief summary concerning the Breast Cancer Clinical Practice Guidelines 2018 edition in English will be helpful for both Japanese and foreign investigators.

## The history of the breast cancer clinical practice guidelines

The “Science-Based Breast Cancer Practice Guidelines” were prepared as a research report on a grant from the Ministry of Health, Labor, and Welfare in 2002 and formed the beginning of the current Breast Cancer Clinical Practice Guidelines. After that, the development of the guidelines was transferred to the Japanese Breast Cancer Society. The first edition was published as five booklets, covering (1) epidemiology and prevention, (2) screening and diagnosis, (3) surgical therapy, (4) radiation therapy, (5) systemic therapy, released in 2004 and 2005. Since then, several revised versions have been published. Previous guidelines were based on scientific evidence such as review data of many clinical trials. Their credibility was based on the “level of evidence (evaluation based on study design)” of the data as described in a previous review [1].

In recent years, in the process of standardization of global guideline development, the procedure for preparing the Breast Cancer Clinical Practice Guidelines has become somewhat outdated. The guidelines up to the previous edition decided on important subjects in daily clinical practice to be posed as clinical questions, comprehensively searched the literature related to this, created the text after critical review of the literature, and recommended it after review by committee members. We have taken the steps to determine the recommended grade and complete the final version. Clinical questions (CQs) with positive results in randomized controlled trials (RCTs) and meta-analyses were often "strongly recommended" for the presence of Level 1 evidence in previous guidelines. Of course, the guidelines for breast cancer treatment up to the previous edition are also highly regarded as largely complete guidelines and it is true that they have contributed significantly to the standardization of breast cancer treatment in Japan. However, there is a point of view that "the balance of benefit and harm" was not adequately covered in the description of the previous guidelines.

## The concept of the 2018 breast cancer clinical practice guidelines

Guidelines are not only a guide to standard practice, but also a tool to provide the reader with the materials, which need to be considered when encountering a problem. Our daily practice is a series of interventions (diagnosis, surgical therapy, radiation therapy, systemic therapy, etc.) and when deciding which method to use, we should be unconsciously choosing means in consideration of benefits and harms. However, when deciding the intervention means, it is important to decide on the intervention means, while sharing the balance of benefits and harms with the patient, as well as the judgment of the individual physician (shared decision making). Therefore, in this revision of the guidelines for breast cancer medical care, we proceeded with the concept of creating guidelines as a tool for doctors and patients to make shared decisions. The concept of the 2018 Breast Cancer Clinical Practice Guidelines is to provide a support tool for both doctor and patient to use shared decision making. Minds Handbook for Clinical Practice Guideline Development 2014 [2, 3] was used as a reference for the preparation of these guidelines.

## Proceeding to make the guidelines

In this 2018 edition, we set multiple outcomes (both in terms of benefits and harms) for each clinical question (CQ) (about 3–6) and determined the clinical importance (1–9) for each outcome. Then, after the literature search/extraction from keywords related to the CQ, a quantitative or qualitative systematic review was conducted for each one of multiple outcomes and the strength of the recommendation for the CQ was then taken into consideration at each small board meeting, regarding the balance between benefit and harm. Finalized recommendations from each session were confirmed through discussion and voting at the recommendation decision meeting, which included doctors, nurses, pharmacists, and patients. Based on this final decision, the responsible committee member wrote a commentary and completed the final version after mutual review.

## Content of the 2018 guidelines

1. BQ (background question)
  Content that is regarded as a standard treatment with old guidelines with which you should comply even if you are not a specialist, and content that should be treated originally as a CQ with content that is routinely lost in clinical judgment, but only old data are included. In addition, any contents that are expected to have no new evidence in the future are also regarded as BQ and outlined.
  ∗Structure: BQ sentence, statement, and commentary.
2. CQ (clinical question)
  The subject of daily clinical questioning is taken up, a quantitative or qualitative systematic review is carried out, proposals are put to a vote at a recommendation decision meeting, the strength of recommendations is decided, and commentary about the recommendations is provided. We comment on points of discussion at the decision meeting.
  ∗Structure: CQ sentence, recommendation, strength of recommendation, consensus rate, strength of evidence and commentary.
3. FQ (future research question)
  Although there is insufficient data on these subjects to be taken up as a CQ, it explains the current thinking about the CQ that is considered to be an important issue in the future.
  ∗Structure: FQ sentence, statement, and commentary.

## Recommendation grade, evidence grade

Recommendation grade is shown in Table 1. This recommendation grade is determined based on the balance between risk and benefit, which occurred by intervention in daily clinical practice, the consistency of patient’s preference, and the economical viewpoint. The strength of the recommendation followed Minds Handbook for Clinical Practice Guideline Development 2014 and is split up into four grades. Rough correspondence with recommendation grade A, B, C1, C2, and D to the previous edition is also indicated. Many CQ are not intervention CQ in epidemiology and prevention field as similar with previous edition. Many questions which should be mentioned in daily life were picked as CQ. Therefore, we show the probability of the scientific basis as the evidence grade without taking a stand, which recommends either do or not (Table 2).

**Table 1:** Recommendation grade

Strength of recommendation	Statement	Clinical meaning	Grade of previous edition
1	Strongly recommend to do	Examination should be carried out	A
2	Weakly recommend to do	Recommended to do based on consideration of the balance between harm and profit, as well as patient’s values	B, C1
3	Weakly recommend to not do	Recommend not to do based on consideration of the balance between harm and profit, as well as patient’s values	C2
4	Strongly recommend to not do	Should not be examined because the harm of the CQ far exceeds the profit	D

**Table 2:** Evidence grades

Convincing	There is enough evidence to determine that an association with cancer risk is certain and taking preventive action is recommended
Probable	There is enough evidence to determine that an association with cancer risk is almost certain and taking preventive action is generally recommended
Limited-suggestive	Although neither ‘‘convincing’’ nor ‘‘probable’’ can be determined, there is evidence suggesting an association with cancer risk
Limited-no conclusion	Data are insufficient and an association with cancer risk cannot be determined
Substantial effect on risk unlikely	There is enough evidence to determine that there is no substantial effect on cancer risk

## Conclusion

A guideline is not an absolute rule book. Even if you explain that an interpretive sentence is read and what this means in terms of recommendation for a patient, the decision of each particular patient may change according to the patient's sense of values. Each valued judgment in such a patient should be respected with the premise that patients are given the right information. We believe that more accurate medical examination and treatment will be spread via the utilization of this guideline at clinical sites, and as a result, many patients would like to gain more benefit in daily practice.
