# Correction to: The Japanese Breast Cancer Society Clinical Practice Guidelines, 2018 edition: the tool for shared decision making between doctor and patient

**DOI:** 10.1007/s12282-021-01253-w

**Published:** 2021-05-19

**Authors:** Hiroji Iwata, Shigehira Saji, Masahiko Ikeda, Masashi Inokuchi, Takayoshi Uematsu, Tatsuya Toyama, Rie Horii, Chikako Yamauchi

**Affiliations:** 1 Department of Breast Oncology, Aichi Cancer Center Hospital, 1-1, Kanokoden, Chikusa-ku, Nagoya, Aichi 464-8681 Japan; 2 Department of Medical Oncology, Fukushima Medical University, Fukushima, Japan; 3 Department of Breast and Thyroid Surgery, Fukuyama City Hospital, Fukuyama, Japan; 4 Department of Breast Endocrine Surgery, Kanazawa Medical University, Ishikawa, Japan; 5 Department of Breast Imaging and Breast Intervention Radiology, Shizuoka Cancer Center, Shizuoka, Japan; 6 Department of Breast Surgery, Nagoya City University Graduate School of Medical Sciences, Nagoya, Japan; 7 Department of Pathology, Saitama Cancer Center, Ina, Japan; 8 Department of Radiation Oncology, Shiga General Hospital, Moriyama, Japan

## Correction to: Breast Cancer (2020) 27: 1–3 https://doi.org/10.1007/s12282-019–01021-x

The article “The Japanese Breast Cancer Society Clinical Practice Guidelines, 2018 edition: the tool for shared decision making between doctor and patient”, written by Hiroji Iwata, Shigehira Saji, Masahiko Ikeda, Masashi Inokuchi, Takayoshi Uematsu, Tatsuya Toyama, Rie Horii, Chikako Yamauchi, was originally published electronically on the publisher’s internet portal on 2 April 2020 without open access. After publication in volume 27, issue 1, page 1–3, the author decided to opt for Open Choice and to make the article an Open Access publication. Therefore, the copyright of the article has been changed to © The Author(s) and the article is forthwith distributed under the terms of the Creative Commons Attribution 4.0 International License, which permits use, sharing, adaptation, distribution and reproduction in any medium or format, as long as you give appropriate credit to the original author(s) and the source, provide a link to the Creative Commons licence, and indicate if changes were made.

The images or other third party material in this article are included in the article’s Creative Commons licence, unless indicated otherwise in a credit line to the material. If material is not included in the article’s Creative Commons licence and your intended use is not permitted by statutory regulation or exceeds the permitted use, you will need to obtain permission directly from the copyright holder.

To view a copy of this licence, visit http://creativecommons.org/licenses/by/4.0/.

The original article has been updated.

